# Development of a Family Engagement Measure for the Intensive Care Unit

**DOI:** 10.1016/j.cjco.2022.07.015

**Published:** 2022-08-05

**Authors:** Michael Goldfarb, Sylvie Debigaré, Nadine Foster, Nataliya Soboleva, France Desrochers, Laura Craigie, Karen E.A. Burns

**Affiliations:** a Division of Cardiology, Jewish General Hospital, McGill University, Montreal, Quebec, Canada; b Patient and Family Partnership Committee, Canadian Critical Care Trials Group, Markham, Ontario, Canada; c Division of Critical Care, University of Calgary, Calgary, Alberta, Canada; d Department of Nursing, Jewish General Hospital, McGill University, Montreal, Quebec, Canada; e Department of Nursing, McGill University Health Centre, McGill University, Montreal, Quebec, Canada; f Interdepartmental Division of Critical Care, Department of Medicine, University of Toronto, Toronto, Ontario, Canada; g Li Ka Shing Knowledge Institute, Toronto, Ontario, Canada; h Unity Health Toronto—St. Michael’s Hospital, Toronto, Ontario, Canada; i Department of Health Research Methods, Evidence, and Impact, McMaster University, Hamilton, Ontario, Canada

## Abstract

**Introduction:**

Family engagement is a goal of care delivery in the intensive care unit (ICU). However, currently, no validated instrument for the ICU is designed specifically to measure family engagement. Our objective was to develop a novel family engagement measure.

**Methods:**

The **Fam**ily **E**ngagement (FAME) tool was developed through an iterative process, with input from experts, family members, and end-users. The FAME questionnaire is composed of 12 items. Each item is scored using a 5-point Likert scale and transformed onto a 0-100-point range, with higher scores indicating greater engagement. We performed a single-site pilot study for family members of patients in a cardiovascular ICU.

**Results:**

The FAME tool had a high construct validity and required an average of 3.33 minutes to complete. A total of 32 family members completed the FAME questionnaire (mean age: 52.4 ± 14.2 years; 71.4% female; 47% adult child ; 31% spouse/partner). The overall mean FAME score was 84.0% ± 25.2%. Differences in engagement across various domains were identified.

**Conclusions:**

The FAME measure is a focused and pragmatic tool to measure the degree and type of family engagement in care of patients in the ICU. Further studies are needed to evaluate the FAME tool in a larger population.

## Résumé

**Introduction:**

La participation de la famille est un objectif établi dans la prestation de soins dans les unités de soins intensifs (USI). Pourtant, il n’existe actuellement aucun instrument validé conçu précisément pour mesurer la participation des familles dans les USI. Notre objectif était d’élaborer une nouvelle façon de mesurer la participation des familles.

**Méthodologie:**

L’outil **Fam**ily **E**ngagement (FAME; participation de la famille) a été conçu par un processus itératif, avec la collaboration d’experts, de membres de familles et d’utilisateurs des services. Le questionnaire FAME comporte 12 éléments. Chaque élément est noté à l’aide d’une échelle de Likert en 5 points et transposé en un score de 0 à 100, un score plus élevé indiquant une plus grande participation. Nous avons réalisé une étude pilote dans un seul établissement auprès des membres de la famille de patients d’une USI en cardiologie.

**Résultats:**

L’outil FAME avait une bonne validité conceptuelle et nécessitait en moyenne 3,33 minutes à remplir. Au total, 32 membres de la famille ont rempli le questionnaire FAME (âge moyen : 52,4 ± 14,2 ans; 71,4 % de femmes; 47 % d’enfants adultes; 31 % de conjoints). Le score FAME moyen global a été de 84,0 % ± 25,2 %. Des différences quant à la participation aux divers volets ont été relevées.

**Conclusions:**

Le questionnaire FAME est un outil ciblé et pratique qui permet de mesurer le degré et le type de participation de la famille dans les soins des patients séjournant à l’USI. D’autres études doivent être menées pour évaluer l’outil FAME au sein d’une population élargie.

Family members are a valuable resource for contemporary healthcare delivery in the intensive care unit (ICU). Family may be engaged in communicating and making decisions with the healthcare team, providing emotional or physical support to their loved one, and actively contributing to care delivery. “Family” is typically defined broadly as anyone with a biological, legal, or emotional relationship with the patient that the patient would like to be involved in her or his care.^1^

Families increasingly expect to be informed about and participate in the care process.^2^ A survey of 544 family members in 78 ICUs in France found that 88% of family members felt that participation in care should be offered to families.^3^ Growing evidence indicates that engaging families in care leads to improved patient, family, and clinical outcomes.^4^ As a result, professional society guidelines recommend engaging families in patient care as part of standard ICU practice.^5^^,^^6^

Despite increasing recognition of the importance of patient and family engagement in ICU care, currently, no validated instruments are available that are designed specifically to measure family engagement in the adult critical care setting. The lack of a standardized measurement tool has hindered the ability of researchers and healthcare organizations to quantify the impact of interventions on improving engagement in care and has limited the evaluation of the relationship between family engagement and outcomes. Recent society statements pertaining to family engagement have highlighted the strong need for development of a validated measure of family engagement in acute care.^7^

In collaboration with a multidisciplinary team, we sought to develop a novel measure to quantify family engagement in the ICU setting. The objective of this study is to describe the development of the FAMily Engagement (FAME) measure for the critical care context.

## Methods

We used a systematic approach to develop, test, and administer the FAME tool described by Hamzeh and colleagues (Questionnaire Origin and Development Appraisal tool).^8^ The FAME tool was developed through an iterative and collaborative process by an interdisciplinary group with expertise in person-centered ICU care (M.G., K.B.), survey methodology (K.B.), ICU nursing (F.D., L.C.), and patient and family partners (S.D., N.F.).

### Literature review

We reviewed the literature (published, grey) for tools evaluating healthcare engagement of families in the ICU. We performed a targeted search of the literature from 1980 to 2021, using combinations of key words to identify relevant studies. Search-term categories included engagement (engagement, participation, involvement), evaluation tools (measure, instrument, tool), critical care setting (intensive care unit, critical care), and family (family, family member, caregiver).

### Tool development process

#### Item generation and reduction

Members of our multidisciplinary team generated questionnaire items using the following: (i) family engagement domains described by Olding and colleagues; and (ii) key principles of family-centered care listed by the Institute for Patient and Family-Centered Care.^9^, ^10^, ^11^ Family engagement domains included family presence, family needs, communication/education, decision-making, and direct contribution to care. Family-centered care principles included dignity and respect, information sharing, participation, and collaboration. To decrease respondent burden, we reduced the number of items within domains pertaining to the overlap that existed between family engagement domains and family-centered care principles. Aligned with health literacy recommendations, the questionnaire and accompanying instructional texts were written at a 6^th^-to-8^th^-grade reading level.^12^ The final version of the questionnaire was formatted at a 6^th^-grade reading level using the Flesch-Kincaid Grade Level Index.

#### Content and face validity assessment

**Figure 1:**
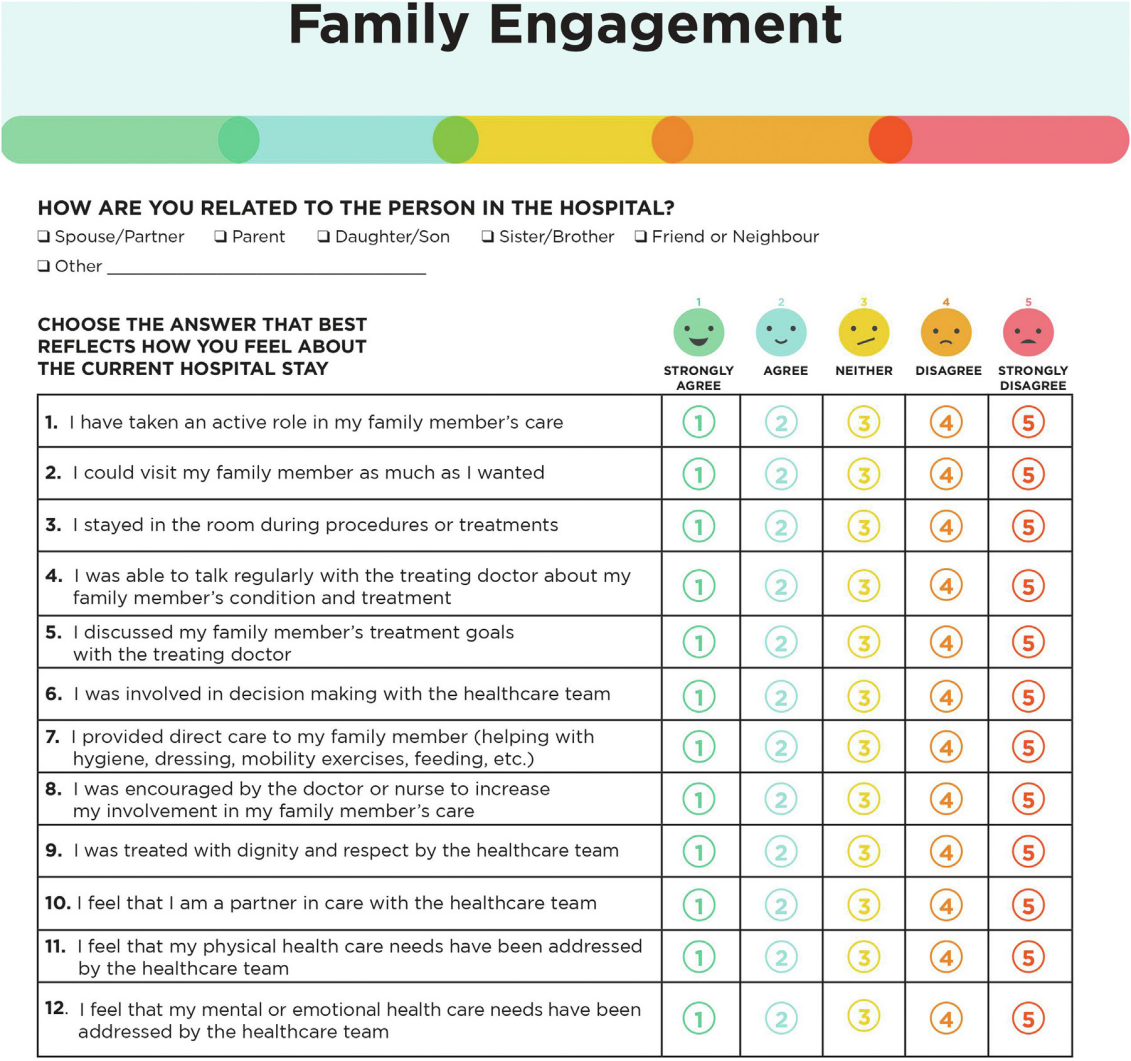
The **Fam**ily **E**ngagement (FAME) measure.

Pilot-testing of the English and French versions of the questionnaire was performed with 6 individuals (a patient**,** a clinical nurse specialist, **a** cardiac ICU nurse manager, a quality-of-care expert, and 2 family representatives) to identify poorly worded, redundant, or irrelevant items. We then assessed the clinical sensibility (clarity and content validity) of the questionnaire with a physician, a nurse research coordinator, and 3 members of the general public. We revised the questionnaire after each phase of testing based on respondent feedback. We ensured that the final version of the questionnaire included each of the engagement categories identified by Olding, and the family-centered care principles (Table 1). The instrument showed moderate to high content validity of individual items (I-content validity index [CVI] range 0.70-1.00), as well as high overall content validity (S-CVI/universal agreement = 0.82; S-CVI/average =  0.92).^13^ The final questionnaire was 1 page in length and was printed in color (Fig. 1). An identical-looking electronic version of the questionnaire was subsequently created. The study, entitled “Family Engagement in Acute Cardiovascular Care” was approved by the Jewish General Hospital’s Quality Program. No institutional review board review was necessary because the study did not fall under the board's guidelines as human subjects research. Procedures were followed in accordance with the ethical standards of the responsible institutional committee on human experimentation and with the Helsinki Declaration of 1975.

**Table 1:** Questionnaire items and domains assessed

Items	Domain assessed
1. I have taken an active role in my family member’s care	Perception of current engagement; active role in care
2. I could visit my family member as much as I wanted	FE domain: family presence
3. I stayed in the room during procedures or treatments	FE domain: family presence
4. I was able to talk regularly with the treating doctor about my family member’s condition and treatment	FE domain: communication/education
5. I discussed my family member’s treatment goals with the treating doctor	FE domain: communication/education
6. I was involved in decision making with the healthcare team	FE domain: decision-making
7. I provided direct care to my family member (helping with hygiene, dressing, mobility exercises, feeding, etc.)	FE domain: direct care
8. I was encouraged by the doctor or nurse to increase my involvement in my family member’s care	Perception of current engagement practices: health system
9. I was treated with dignity and respect by the healthcare team	FCC domain: dignity and respect
10. I feel that I am a partner in care with the healthcare team	FCC domain: partnership
11. I feel that my physical healthcare needs have been addressed by the healthcare team	FE domain: family needs
12. I feel that my mental or emotional healthcare needs have been addressed by the healthcare team	FE domain: family needs

FCC, family-centred care; FE, family engagement.

#### Questionnaire composition and scoring

The FAME tool was composed of 12 items, reflecting the following domains: perception of engagement (questionnaire item [Q]1, Q8); family presence (Q2, Q3); communication/education (Q4, Q5); decision-making (Q6); contributing to care (Q7); family-centered care (Q9, Q10); and family needs (Q11, Q12). A 5-point Likert scale (1 = strongly agree; 2 = agree; 3 = neither; 4 = disagree; 5 = strongly disagree) is used for responses to individual questions within domains. Scale scores are transformed onto a 0-100-point scoring system, with higher and lower scores indicating greater and lower engagement in care, respectively. In this regard, a Likert-scale score of 1 (strongly agree) for an individual question reflects an engagement score of 100, and a Likert-scale score of 5 (strongly disagree) aligns with an engagement score of 0. An overall FAME engagement score (range 0-100) is calculated by adding up the scores (numerator) and dividing by the number of questions answered (denominator).

#### Pilot-testing

We performed a single-site pilot feasibility study for family members of people admitted to the cardiovascular ICU at the Jewish General Hospital, an academic tertiary care hospital in Montreal, Canada. We distributed the FAME tool to family members who were present at the bedside on 3 nonconsecutive days. Family members were invited to complete the paper questionnaire or the electronic version through provision of a QR (for quick response) code. Only one family member per patient was eligible to complete the FAME tool. Participation was voluntary. The pilot study was approved as a quality-improvement project by the quality department at the participating institution.

## Results

In total, 35 family members were approached to request participation. Of these, 2 family members declined to participate, as they felt overwhelmed, and 1 family member was excluded due to a language barrier. A total of 32 family members of patients admitted to the cardiovascular ICU completed the FAME tool (mean age: 52.4 ± 14.2 years; 71.4% female; 26 via written response; 6 via electronic response). Relationships of respondents to patients included adult child (47%), spouse or partner (31%), siblings (10%), parents (6%), and friends or neighbours (6%). All respondents (N = 32) completed each of the 12 questions of the questionnaire.

**Figure 2:**
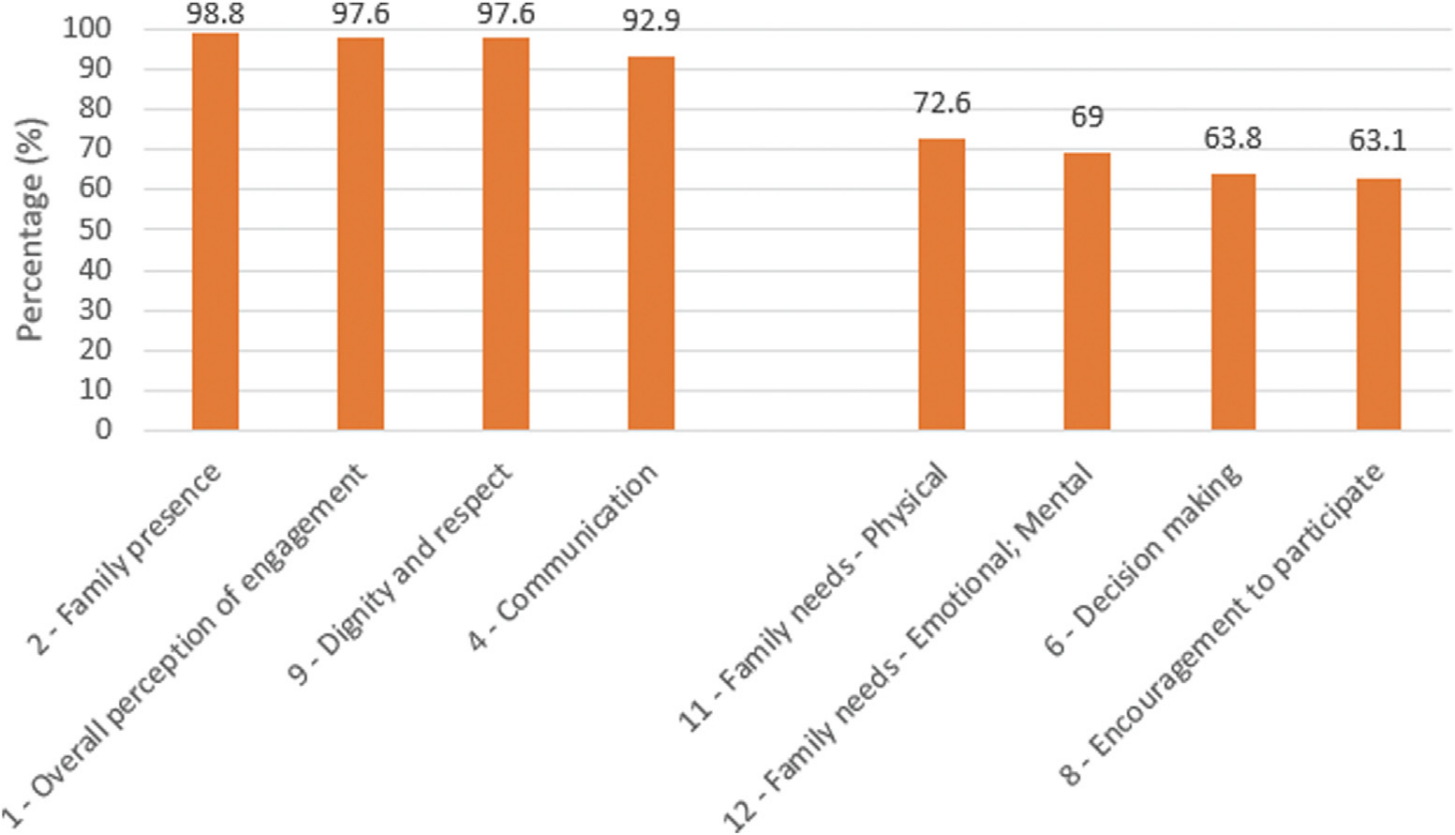
Highest and lowest engagement scores. The 4 items with the highest scores are on the left side of the chart, and the 4 items with the lowest scores are on the right side of the chart. Numbers indicate questionnaire item number.

The overall mean FAME score was 84.0% ± 25.2%. The mean questionnaire completion time was 3 minutes, 20 seconds. In descending order, the highest FAME engagement scores in our cohort reflected engagement through family visitation (98.8% ± 5.5%), overall perspective on engagement (97.6% ± 7.5%), and receiving information (92.9% ± 17.9%; Fig. 2). Family members felt strongly that they were treated with dignity and respect (97.6% ± 10.9%). The lowest engagement scores reflected encouragement for family members to participate in their loved one’s care (63.1% ± 31.2%) and in shared decision-making (63.8% ± 30.9%).

## Discussion

The FAME tool was developed with input from experts, patients and family members, and end-users. We identified differences in engagement across various domains of care, especially those reflecting engagement in patient care and shared decision-making. We showed that the FAME tool has high construct validity and can be completed in 3.33 minutes by family members.

Current approaches to measuring family engagement are limited; these include proxy measurements of engagement and measurements focused on selected aspects of engagement. The Center for Medicaid Services in the US has proposed process “metrics” to evaluate family engagement, including “shift-change huddles” involving patients and families, identification of an accountable engagement program leader, family representation on hospital governance committees, and quality-improvement teams. In addition, the Center for Medicaid Services highlights the need for shared decision-making, patient activation, health literacy surveying, medication management, and support to help ensure that patient and family voices are heard.^14^ However, these factors could be considered “strategies” to engage families rather than “metrics” for engagement, as no tool or method is currently available that is designed to score or benchmark family engagement.

Several tools have been developed to measure aspects of engagement. The Caregiving Health Engagement Scale was developed to assess family caregivers’ psychosocial experience of engagement in their loved one’s healthcare.^15^ However, this instrument does not capture the practical aspects of family-member participation in care, which is necessary to quantify family engagement in the ICU setting. In addition, measurement instruments are validated for a specific population, purpose, and care context. Qualitative differences exist in family members’ ability to interact with patients in the ICU, as compared to other settings. The Family Satisfaction in the ICU scale is a self-reported instrument to assess family satisfaction with ICU care after hospital discharge of the patient.^16^ This scale includes selected elements of engagement, such as communication, education, and decision-making, but it does not include other domains, such as family presence and participation in direct care. With this instrument, aspects of family engagement are measured after patient hospitalization, as though they are outcome measures as opposed to process measures. Thus, with this scale, engagement is considered to be the end result of care and not a means to achieve a desired outcome.

Although the Critical Care Family Needs Inventory includes some elements of engagement, such as information sharing and communication, it is complex to administer, rendering it impractical for routine clinical or administrative use. Moreover, this instrument was not developed primarily to measure family engagement in care.^17^ By contrast, FAME is a pragmatic and focused tool that includes all operational domains of family care engagement for the ICU. The FAME tool is administered during hospitalization to quantify the engagement in the care process in real time. As a result, it can be used to capture process metrics that guide research, support evidence-based practices, systematize improvement efforts, identify specific areas for intervention, and reduce variation in care delivery.

Data are limited regarding aspects of engagement that are considered to be important to family members.^18^ The FAME tool can identify domains of engagement in care that are lacking. In our pilot-testing, the domains with the highest level of engagement were family visitation and receipt of information, whereas the lowest scores reflected family engagement in shared decision-making and participation in care. Shared decision-making in the ICU environment has been identified previously as a particular area of family member concern.^6^ Identification of these deficient areas in real time could guide interventions by the clinical team to improve the process of family engagement in the ICU.

### Implications and future directions

The FAME questionnaire was developed and tested at an urban hospital serving a catchment area with an enormous diversity of languages, countries of origin, and ethnic backgrounds. Despite this diversity, only one family member was excluded in our study because of an inability to complete the questionnaire in English or French. Other geographic locations or healthcare settings may have a higher percentage of people who are not able to complete the FAME questionnaire in English or French. Thus, adaptation of the FAME questionnaire for other languages and cultures is needed.

The FAME tool requires validation in a large, multicentre cohort. Once validated, the relationship between engagement, as measured by the FAME tool score, and relevant patient, family, and clinical outcomes can be further explored. For example, the FAME tool could be used to provide real-time feedback on family engagement to the clinical team and to trigger targeted interventions. Quantifying family engagement and its components may guide quality-improvement efforts and provide a comparative process metric for research.

### Limitations

Our study has several limitations. First, the FAME tool was pilot-tested on a small number of family members in a single cardiovascular ICU. As such, the mean overall score and engagement domains may not be reflective of the broader ICU population. To increase its generalizability, further study is needed, with larger populations and more-diverse respondents, to understand the impact of diversity metrics such as age, gender, and race/ethnicity on engagement in care. Second, we cannot rule out selection bias in our cohort, as it included family members who were motivated to participate in this study. To address this concern, we approached consecutive family members over the days that the FAME questionnaire was distributed. Only 2 of 34 family members (5.9%) who were approached declined to complete the questionnaire.
